# Oneida County Veterinary Medical Society

**Published:** 1897-01

**Authors:** J. M. Currie

**Affiliations:** Secretary


Secretary.

### ONEIDA COUNTY VETERINARY MEDICAL SOCIETY.

THE regular quarterly meeting was called to order at 2 P.M., November 3, 1896, in the parlors of Stanwix Hall, Rome, N. Y., by Dr. F. Morrow, President. The following members responded to roll-call: Drs. F. Morrow,

Oneida; L. G. Moore, Trenton; A. Findlay, Camden; Wilson Huff, Rome; and J. M. Currie, Rome.

The minutes of the previous meeting were read and approved. When the order of new business was called, the advisability of this Society issuing certificates to its members was brought up by Dr. Moore; it was well discussed by those present, and finally it was moved by Dr. Huff and seconded by Dr. Moore that a suitable certificate be issued, and that the recipient of such certificate agrees to return the same to the Society whenever he ceases to be in good standing. Carried.

Next came the reading of papers. Dr. Moore read an interesting paper on the "Actions of Barium Chloride and Eserine and Pilocarpine in the Treatment of Impaction of the Intestines in the Horse." He strongly recommended the latter remedy, as a result of his personal experience with them. A lively and interesting discussion followed, and the writer was tendered a vote of thanks for his valuable contribution.

The remaining time was taken up discussing points of interest in our daily practice.

There being no further business to come before the meeting, an adjournment was taken, to meet again the first Tuesday in February, 1897. All departed for their respective homes well pleased with the manner in which the afternoon had been spent.

J. M. Currie, V.S.,
Secretary,

